# Molecular Characterization of Tb, a New Approach for an Ancient Brucellaphage

**DOI:** 10.3390/ijms10072999

**Published:** 2009-07-03

**Authors:** Cai-Zhong Zhu, Hong-Yan Xiong, Jing Han, Bu-Yun Cui, Dong-Ri Piao, Ya-Fei Li, Hai Jiang, Qian Ren, Xiang-Yu Ma, Ya-Ming Chai, Xia Huang, Hong-Yan Zhao, Lan-Yu Li

**Affiliations:** 1 Department of Epidemiology, College of Preventive Medicine, Third Military Medical University / Gaotanyan road 30, Shapingba district, Chongqing 400038, China; E-Mails: zhucaizhong@tmmu.edu.cn (C.-Z.Z.); liyafei1972@sina.com (Y.-F.L.); renqian1973@sina.com (Q.R.); maxiangyutmmu@sina.com (X.-Y.M.); chaiyamingtmmu@sina.com (Y.-M.C.); zlincon@sina.com.cn (X.H.); 2 State Key Laboratory for Infectious Disease Prevention and Control, National Institute for Communicable Diseases Control and Prevention, China CDC / Liu zi No.5, Changping district, Beijing, 102206, China; E-Mails: piaodongri2008@sina.com (D.-R.P.); jianghaicdc@sina.com (H.J.); caizhongzhu2004@yahoo.com.cn (H.-Y.Z.); caizhongzhu2004@yahoo.com.cn (L.-Y.L.); 3 Institute of Combine Injury, School of Preventive Medicine, State Key Laboratory of Trauma, Burns and Combined Injury, Third Military Medical University / Gaotanyan road 30, Shapingba district, Chongqing 400038, China; E-Mail: hanjing8107@sina.com (J.H.)

**Keywords:** brucellaphage, molecular characterization, random amplified polymorphic DNA

## Abstract

Tb (*Tbilisi*), the reference Brucellaphage strain, was classified as a member of the Podoviridae family with icosahedral capsids (57 ± 2 nm diameter) and short tails (32 ± 3 nm long). Brucellaphage DNA was double stranded and unmethylated; its molecular size was 34.5 kilobase pairs. Some sequences were found through RAPD analysis, TA cloning technology, and structural proteins were observed by using SDS-PAGE. Thus, the results have laid the foundation for the wider use of Brucellaphage’s basic mechanisms and practical applications.

## 1. Introduction

The existence of bacteriophage active against brucella was claimed in some works [1–5]. Other researchers were able to isolate brucellaphages such as Tb, Wb, Fi, R, Iz and Np many years ago. Since then, brucellaphages have been used in speciation and biotyping of brucellae, but little is known about the nature of the brucellaphage-host cell relationship, and neither lysogenic phages nor plasmids have been demonstrated, and Brucella phage DNA has not been further characterized. The brucellaphages that have been described differ mainly in the species of Brucella which they infect, and their activity is highly sensitive to smooth-rough variation in their host brucellae. For example *Tbilisi* (Tb) and *Firenze* (Fi) phages replicate in smooth *B. abortus* (and at high concentrations produce “lysis-from-without” of *B. suis*), while *Weybridge* (Wb) replicates in smooth strains of both *B. suis* and *B. abortus. Izatnagar* (Iz) replicates in smooth *B. melitensis, B. suis* and *B. abortus* and has been reported to replicate in some rough brucellae. R/C, a mutant derived from Wb, D and Mc/ 75 phages, lyses the rough species *B. ovis* and *B. canis* as well as some rough strains of other species. Np has a more restricted host range, replicating only in smooth strains of *B. abortus*.

This report describes the reference brucellaphage Tb - an ancient phage with little in-depth study compared with other species of bacteriophage. One of our objectives was to examine the morphology with advanced electronic- microscope technology, and DNA restriction endonuclease profiles of Tb. Finally, we used RAPD (random amplified polymorphic DNA)/AP-PCR (arbitrarily primed PCR) fingerprinting analysis to indicate the difference between Tb and host strain DNA. We hope to thus generate a new scientific understanding of the use of Brucellaphages.

## 2. Results and Discussion

### 2.1. Tb morphology

**Figure 1.**
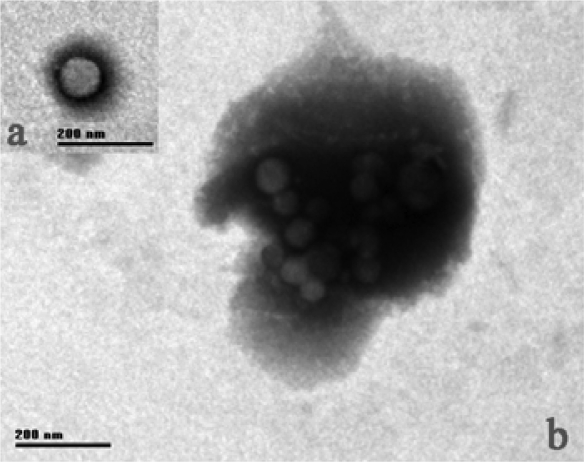
Electron micrograph of phage Tb. (a) Electron micrograph of a phage with a short, stout tail; The polyhedral nature of the viral head is evident. (b) A number of phages which have discharged their contents are still attached to the cocco-bacillus cell.

The virulent phage Tb has an icosahedral capsid of 57 ± 2 nm in diameter, with a collar of 12 ± 2 nm, and a short noncontractile tail 32 ± 3 nm long. The tail has a baseplate of 15 x 3 nm to which a tail fiber of 20 x 2 nm is attached (Figure 1). The phage was classified as a member of the Podoviridae family and it has been observed in less than 1% of known phages [6,7].

### 2.2. Nucleic acid characterization

**Figure 2.**
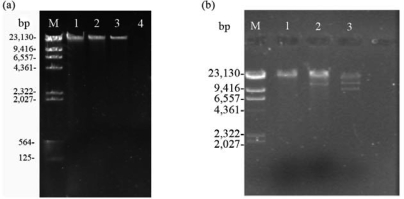
Agarose gel electrophoresis of DNA of Tb. (a) Agarose gel electrophoresis of DNA of Tb(lane 1), digested with nucleases S1 - (lane2), Rnase A - (lane 3) and Dnase I - (lane 4). Molecular size markers (kb) were HindIII-digested lambda DNA. (b) Agarose gel electrophoresis of DNA of Tb (lane 1), digested with restriction endonucleases *Bam*HI (lane 2), *Eco*RI (lane 3). Molecular size markers (kb) were HindIII-digested lambda DNA.

All phage nucleic acid samples were sensitive to Dnase I, but were resistant to digestion by Rnase A and S1 nuclease (Figure 2a). It was concluded that all extracts contained phage with double-stranded linear DNA. The genome size of phage Tb was approximately 34.5 kb. The molecular mass of Tb DNA determined by the sum of the sizes is about 22.4 x 10^6^ daltons (Table 1). This was determined by adding the molecular sizes of *Eco*RI or *Bam*HI -digested fragments of Tb DNA (Figure 2b). The restriction endonuclease profiles were highly reproducible and consistent with phage Tb.

### 2.3. RAPD analysis for identification between phage Tb and host strain B. abortus S 19

**Figure 3.**
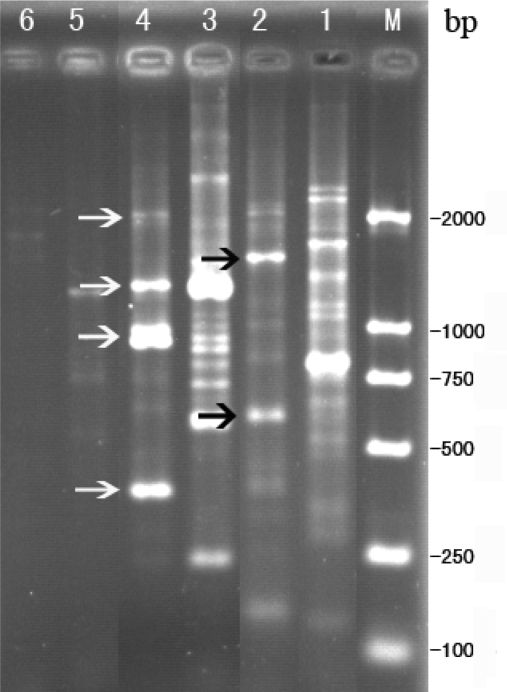
RAPD profile amplified for phage Tb and host strain *B. abortus* S 19. RAPD profile amplified with primer S2, S57 and S59 for phage Tb (lanes 2, 4, 6 respectively for S57, S59 and S2) and host strain B. *abortus* S 19 (lanes 1, 3, 5 respectively for S57, S59 and S2). Arrows on the left indicate distinct bands. The M lane is DL2000 marker (BBI) with each fragment size showing on the right.

Randomly amplified polymorphic DNA (RAPD) profiles were generated with three randomly designed 10-mers, each used separately as an amplification primer. Depending on the primers, the analysis of RAPD profiles resulted in different levels of discrimination between phage Tb and host strain *B. abortus* S 19. In the analysis of phage Tb and *B. abortus* S 19 isolates by RAPD using S2 primer, no obvious band was obtained (Figure 3), so S2 primer should not be counted as means of identification for finding the different characteristics between phage Tb and host strain S19. When using S57 and S59 primers for RAPD, respectively two and four distinct band profiles were obtained for phage Tb. The bands ranged in size from 100 to 2,000 bp. These differences suggest that S57 and S59 primers are essential for a reliable evaluation of the genetic variation between phage Tb and host strain *B. abortus* S 19.

### 2.4. Sequence analysis for RAPD products of phage Tb

**Figure 4.**
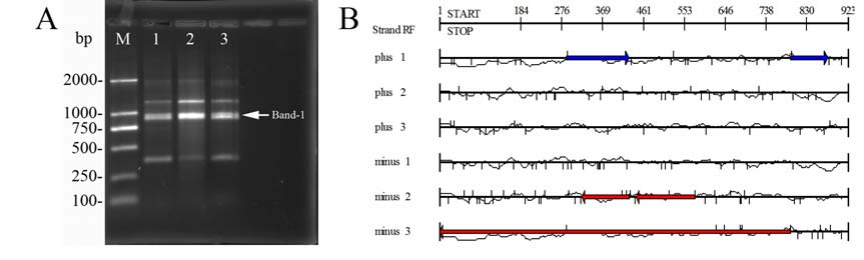
DNA sequence of band-1 sequences. (A)Arrow on the right indicates sequenced band-1 (923bp). The M lane is DL2000 marker (BBI) with each fragment size showing on the left. (B)The blue arrows and red arrows show the putative ORFs (open reading frames).

The TA clone DNAs which obtained from the products of S59 primer amplified phage Tb genomic DNA were sequenced. Figure 4 shows the DNA sequences of band-1. Table 2 summarizes the DNA amplified by S59 primer from phage Tb. DNA product of interest was successfully re-amplified and cloned. BLAST programs were used to compare each of the DNA sequence to nucleotide and amino acid sequences residing in the Basic Local Alignment Search Tool (BLAST) network service and the non-redundant nucleotide sequence database (GenBank + EMBL + DDBJ + PDB), and the nonredundant peptide sequence database (GenBank CDS translations + PDB + SwissProt + SPupdate + PIR) (NCBI) [8].

The nucleotide sequence of the partial DNA band-1 was similar to that of Microcystin-dependent protein-like from *Mesorhizobium* sp. BNC1 (Table 2) and putative phage tail Collar Domain from *Bradyrhizobium* sp. BTAi1. At the amino acid level, band-1 shared 79% identity with Microcystin-dependent protein-like from *Mesorhizobium* sp. BNC1 and 71% identity with putative phage tail Collar Domain from *Bradyrhizobium* sp. BTAi1. Another result from BLAST shows this sequenced band-1 was identified with phage tail sequences of some gram- negative bacterial such as phage Tail Collar Domain family of *Stigmatella aurantiaca* DW4/3-1 and phage Tail Collar of *Kordia algicida* OT-1. According to the research of conserved amino acid motif, structural feature or limited homology, the function of this sequence was speculated for prophage genes and phage related functions such as phage tail collar for host recognition mechanism [9,10] or lysozyme like activities [11–14]. We could thus get some information suggesting that the genome of Tb phage was similar to that of Gram-negative bacteria phage, and putatively determine that the taxonomic position of brucella and its phage was similar to the *Mesorhizobium* sp. BNC1 or *Bradyrhizobium* sp. BTAi1.

### 2.5. Structural protein of phage Tb

**Figure 5.**
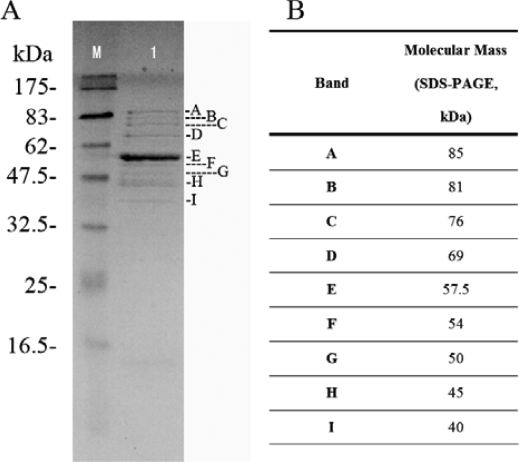
Structural proteins of phage Tb (lane 1) was resolved by SDS polyacrylamide gel electrophoresis, and stained with Coomassie blue. (A)The sizes (in kDa) of the proteins in the broad-range molecular mass standard (lane M) are indicated on the left. (B) Identification of phage Tb proteins from corresponding bands shown in panel A. Letters on the right indicate bands estimated molecular mass by SDS-PAGE.

The protein composition of phage Tb was characterized by SDS-PAGE, which gave rise to nine Coomassie-stained bands of structural proteins (Figure 5, bands A to I). Most gel bands contained more than one protein. The most prominent bands, which likely represent the major capsid proteins, were 57.5 kDa, and 45 kDa (band-E, and band-H) in phage Tb, respectively.

### 2.6. Discussion

Phages are the most abundant organisms in the biosphere, exceeding bacteria by at least one order of magnitude [15]. It is thus not surprising to find virulent Brucella phages worldwide. Since Brucellae is an intracellular pathogen, lysogeny would seem to be a possible mechanism for phage survival in nature but convincing evidence has not yet been obtained [16,17]. We began these studies with the expectation that DNA analytical technique would provide evidence for a lysogenic relationship of phage Tb.

As an international reference strain, Tbilisi is a typical brucellaphage in terms of its morphology, host range, and resistance to chemical and physical agents, differing from other brucellaphages mainly in its host specificity [18]. The results of our brucellaphage characterization studies were in general agreement with those of previous studies [7,18–20].

Our finding that the restriction endonuclease profiles of Tb are identical and very similar to those brucellaphages provides further support for the suggestion that all brucellaphages are members of the same species, though they come from such disparate sources as Russia (Tb), Italy (Fi), England (Wb), India (lz) and Canada (Np).

We describe in this report, the genomic fingerprinting of brucellaphage Tb and host strain B. *abortus* S 19, by amplifying the genomic DNA with single arbitrary primers. The RAPD employed low-temperature stringency to allow sampling of diverse portions of Tb genome without any apparent bias.

The PCR with random oligos is capable of producing unique genetic fingerprints from closely related organisms and this attribute has been previously utilized to differentiate several groups of parasites in trypanosomatid [21–26], coccidial [27,28] or schistosome parasites [29]. And this method was used to increase discrimination in the epidemiological analysis of Salmonella enteritidis strains [30]. We obtained distinct and reproducible pattern of amplified DNA fragments with various arbitrary primers. Phage Tb produced categorically divergent PCR profiles compared to host strain B. *abortus* S 19. The pattern of amplified fragments obtained maybe used to discriminate the variation of brucellaphages.

As RAPD analysis shows that some primers such as S57 and S59 primers are essential for a reliable evaluation of the genetic variation between phage Tb and host strain *B. abortus* S 19. RAPD products were sequenced by TA cloning and the nucleotide sequence of the partial DNA band-1 was similar to putative phage tail collar domain from *Bradyrhizobium* sp. BTAi1. As we know, these sequences were the world’s first findings on biomolecular information of Brucellaphage, and they would open a new era by using modern molecular technologies in brucellaphage research.

Over the course of evolution, bacteriophages, the viruses of eubacteria, have developed unique proteins that attach to and inactivate (or redirect) critical cellular proteins in bacteria, shutting off key metabolic processes to divert host’s metabolism to the production of progeny phages [31]. Maybe we could find a novel mechanism of phage genome regulation of antibiotic sensitivity. And therefore, information in bacteriophage genome could be extracted and used to identify particularly susceptible proteins in their bacterial hosts and to prioritize these targets for anti-Brucella drug discovery. Of course, the conclusions derived from this study have to be corroborated in further research.

In fact, many of human’s deadly bacterial epidemics were and are a by-product of bacteria infecting other bacteria with their viral phages. It is when such an attack does not kill, cripple or maim that antibiotic-resistant pathogens are born for mankind to cope with [32]. And the proper way to deal with this is to learn from nature, how bacteria kill one another, and to recruitrelatively benign bacteria to deliver lytic phage, thereby destroying otherwise incurable infection through lysogeny.

The implications of the novel technique to kill intracellular pathogenic bacteria that was presented are broad and include all classes of intracellular bacteria for both man and animals. It is intended to be used parenterally. Maybe our results suggested the possibility of the new antibiotic target in Brucella to control the spread of Brucellosis.

## 3. Materials and Methods

### 3.1. Phage and bacteria

*Brucella* phage was propagated in the following bacterial host strain: *B. abortus* S 19 (biotype 1) for *Tb*. Tb phage obtained from Weybridge (Ministry of Agriculture, Fisheries and Food Central Veterinary Laboratory, Weybridge, Surrey, U.K.) had originally been prepared at our laboratory. The host propagating strain that had accompanied the phage will be referred to as strain *B. abortus* S 19.

### 3.2. Propagation of Brucella phages

Phage stocks were prepared either by the agar double-layer technique [33] as modified for brucella-phage by McDuff, Jones & Wilson [34], or in stirred brucella cultures grown in *Albimi brucella* broth [35]. In both methods a smooth culture of *B. abortus* S 19 in the logarithmic phase was used as inoculum. Standard methods of species identification, as outlined by Jones [36], showed the bacterial culture to be a non-CO_2_-requiring B. *abortus* of smooth-intermediate colonial morphology. As the culture was somewhat unstable, it was necessary to re-isolate smooth intermediate colonies to maintain phage susceptibility.

Two methods of bacteriophage concentration were assessed: polyethylene glycol (PEG) precipitation and ultracentrifugation. The former was modified from Yamamoto *et al*. (1970)[37], where 10% (w/v) PEG 6000 was added to the filtered lysate followed by filtration through 0.22 μm membrane filters (Oxoid, London, U.K.) and gently mixed to dissolve. The lysates were incubated at 4 °C for 60 min and the precipitated particles pelleted by spinning at 8,000 *g* for 10 min. Pellets were resuspended in 0.01 original volume sterile SM buffer (5.8 g sodium chloride, 2 g magnesium sulphate, 100 mg gelatin, 50 mL 1 mol·L^−1^ Tris (pH 7.5) and 945 mL distilled water). The ultracentrifugation method was modified from that of H.J. Oakey [38], where filtered extracts were ultracentrifuged at 200,000 *g* for 4 h. Pellets were resuspended in 0.01 original volume sterile SM buffer. Aliquots of 100 μL were removed for transmission electron microscopy (TEM) and the remaining concentrates were stored at 4 °C.

### 3.3. Nucleic acid extraction from the isolated bacteriophage

This method was based upon that described by Sambrook and Russell [39]. Bacteriophage from the concentrated solutions were lysed with the addition of EDTA (final concentration 20 mmol·L^−1^), proteinase K (final concentration 50 μg·mL^−1^) and SDS (final concentration 0.5%) and incubation at 56 °C for 1 h. The nucleic acid was purified by a phenol, a phenol/chloroform and a chloroform extraction. This method was modified, whereby the phenol extractant was heated to ~60 °C, added to the lysate, mixed by inversion, incubated at 60 °C for 30 min, centrifuged at 5,000 *g* for 10 min to separate the phases and the aqueous phase was removed for the next extraction. The final aqueous phase was dialysed overnight against TE. In this method, nucleic acid yield was estimated through agarose gel electrophoresis and comparison of ethidium bromide stain intensity with known DNA standards (Hind *III* cut lambda phage DNA; New England Biolabs, Ipswich, MA, USA).

### 3.4. Electron microscopy

All electron micrographs were obtained from phage particles purified by density-gradient centrifugation. They were diluted 1:100 in sterile water, and small drops were placed on carbon coated mica fragments. The mica carriers were dipped into a drop of 2% uranyl acetate or 2% ammonium molybdate in water, and the carbon film carefully floated. Suitable 400 mesh grids (G400Hex-C3-Grid; SCI, Munich, Germany) coated with a Colodion-Film (prepared from 2% colodion in amyl acetate) were used to absorb the carbon film fragments with phage particles to the grid. After air-drying for 10 min, grids were directly used for transmission electron microscopy (Philips CM100; acceleration voltage 100 kV). Images of negatively stained phage particles were taken using a TVIPS Fast scan CCD camera; magnification was between 20,000 and 39,000 ×.

### 3.5. Nucleic acid characterization

The nucleic acid extracts were diluted to a standard concentration of ~20 ng·μL^−1^. Each extract was subjected to a digestion with DNase I (Sigma Aldrich), RNase A (Sigma Aldrich), S1 nuclease (Promega). Each digestion was carried out upon ~250 ng nucleic acid. All reactions were terminated by using EDTA (10 mmol·L^−1^ final concentration) and analysed by using 0.8% agarose gel electrophoresis at 5 V cm^−1^.

### 3.6. Restriction endonuclease analysis

*Bam*HI and *Eco*RI were purchased from Promega. About 1 μg of each DNA sample was incubated with approximately 10 units of enzyme for 2 h at 37 °C in the appropriate buffers. For double digestions, DNAs of the primary digests were purified by phenol-chloroform extraction and ethanol precipitation before the secondary cleavages. DNA fragments were resolved by horizontal gel electrophoresis using 0.8% agarose in Tris-borate buffer.

### 3.7. RAPD

RAPD was performed with the genomic DNAs essentially by the method described by Okuda *et al*.(1997) [40] using 3 randomly chosen 10-base random primers (S2, S57, S59) (Table 3) purchased from Sangon Co. (Shanghai, China). PCR reactions were carried out in 0.2 mL thin-walled PCR tubes with a total reaction volume of 25 μL containing 10 mM of Tris–HCl (pH 8.3), 50 mM of KCl, 2 mM of MgCl_2_, 1.5 unit of Taq DNA polymerase, 0.2 mM each of dATP, dCTP, dGTP and dTTP (Bangalore Genei), 0.4 μM of primers and 50 µg genomic DNA. Amplification was programmed for 45 cycles in a PCR thermocycler (Eppendorf version 2.30. 31-09, Germany). Each cycle consisted of a denaturation step at 94 °C for 1 min, a primer annealing at 36 °C for 1 min, a primer extension step at 72 °C for 2 min. The amplified DNA fragments were separated by electrophoresis on 1.5% agarose (Sigma-Aldrich, USA) gel in 1×Tris borate EDTA (89 mM Tris–HCl, 89 mM boric acid and 2 mM EDTA pH 8.0) and were stained with ethidium bromide (0.75 μg/mL) to document using a UV transilluminator (Bio-Rad, Germany). The molecular size of amplified fragments was estimated using a DL2000 marker (Tiangen, Beijing, China).

### 3.8. Subcloning of RAPD products

These purified RAPD products from the PCR with random primers was subcloned according to the Takara T Vector System I (Takara, Dalian, China) to prepare the DNA for efficient and accurate sequencing. PCR product was ligated into the pMD18-T plasmid vector at 4°C overnight. Ligations were then transformed into DH5α Escherichia coli competent cells (Tiangen, Beijing, China) and grown overnight on LB agar with 100 μg/mL ampicillin and 20 mg/mL Xgal. White colonies (indicating successful ligation) were picked and grown in LB-Amp100 μg/mL broth overnight at 37 °C. The plasmid DNA was harvested using the AxyPrep Plasmid Miniprep Kit, according to protocol (Axygen, CA, USA). The TA clone DNA was then sequenced using M13R(-48) primer(AGC GGA TAA CAA TTT CAC ACA GGA).

### 3.9. Computer-assisted Sequence Analysis

Using a plasmid-specific primer, we carried out sequencing reactions using Prism Ready Reaction Dye Deoxy terminators (Applied Biosystems, Foster City, CA, USA) according to the manufacturer’s instructions. Residual dye terminators were removed from the completed sequencing reaction using a Centri-sep spin column (Princeton Separation, Adelphia, NJ, USA). An Applied Biosystems model 373A DNA Sequencing System was used for sequence analysis. Homology searching was performed at the National Center for Biotechnology Information (NCBI), using the Basic Local Alignment Search Tool (BLAST) network service and the non-redundant nucleotide sequence database (GenBank + EMBL + DDBJ + PDB), and the non-redundant peptide sequence database (GenBank CDS translations + PDB + SwissProt + SPupdate + PIR).

### 3.10. Analysis of Phage Tb Structural Proteins

The purified phage preparation (1 × 10^10^ PFU/mL) and the host strain S19 were then dialyzed and analyzed for structural proteins by standard Tris-glycine 12% SDS-polyacrylamide gel electrophoresis (PAGE) procedures [41]. Samples were mixed with 4× sample loading buffer and boiled for 5 min before loading. A broad range of protein marker (New England Biolabs, Ipswich, MA, USA) was used to estimate molecular masses. Protein bands were stained with Coomassie Brilliant Blue R-250 (Sigma).

## 4. Conclusions

In summary, we present a systematic approach to identify the morphology, molecular and protein characteristics of the reference strain- Tb phage. And our approach allows us to identify and prioritize bacterial proteins that, as far as we know, are not currently pursued by the industry as targets for antibiotic development.

## Figures and Tables

**Table 1.** *Brucella* phage DNA restriction fragment sizes (× 10^6^ daltons).

**Fragment**	***Bam*HI**	***Eco*RI**
A	14.9	9.7
B	7.2	7.1
C		5.89
Total	22.1	22.69
Average	22.4	

**Table 2.** Homology search results for DNAs in response to phage Tb RAPD products amplified by S59 primer.

**TA clone**	**Fragment size (bp)**	**Best hits (blastn or blastx) analysis to nr database**	**General function**	**Score(bits)**	**E Value**	**Accession no.**
Band-1	923	Microcystin-dependent protein-like from *Mesorhizobium sp. BNC1*	Microcystin-dependent protein-like	157	7e-37	gi:110283346
		*Bradyrhizobium sp. BTAi1*	putative phage tail Collar Domain	66.2	3e-09	gi:148251626
		*Stigmatella aurantiaca DW4/3-1*	phage Tail Collar Domain family	55.8	4e-06	gi:115368091
		*Kordia algicida OT-1*	phage Tail Collar	53.5	2e-05	gi:161324659

**Table 3.** List of primers used and their base sequences.

**Sr. no.**	**Primer**	**Sequence**
1	S2	5′- TGATCCCTGG -3′
2	S57	5′- TTTCCCACGG -3′
3	S59	5′- CTGGGGACTT -3′
